# Boolean analysis reveals systematic interactions among low-abundance species in the human gut microbiome

**DOI:** 10.1371/journal.pcbi.1005361

**Published:** 2017-06-22

**Authors:** Jens Christian Claussen, Jurgita Skiecevičienė, Jun Wang, Philipp Rausch, Tom H. Karlsen, Wolfgang Lieb, John F. Baines, Andre Franke, Marc-Thorsten Hütt

**Affiliations:** 1 Computational Systems Biology, Department of Life Sciences and Chemistry, Jacobs University Bremen, Campus Ring 1, D-28759 Bremen, Germany; 2 Institute for Digestive Research, Lithuanian University of Health Sciences, Kaunas, Lithuania; 3 Institute for Experimental Medicine, Christian Albrechts University of Kiel, Kiel, Germany; 4 Max Planck Institute for Evolutionary Biology, D-24306 Plön, Germany; 5 Institute of Clinical Medicine, Faculty of Medicine, University of Oslo, Postbox 1171 Blindern, 0318 Oslo, Norway; 6 Norwegian PSC Research Center, Department of Transplantation Medicine, Division of Cancer Medicine, Surgery and Transplantation, Oslo University Hospital Rikshospitalet, Postbox 4950 Nydalen, 0424 Oslo, Norway; 7 Institute of Epidemiology and Biobank PopGen, Christian Albrechts University of Kiel, Kiel, Germany; Pennsylvania State University, UNITED STATES

## Abstract

The analysis of microbiome compositions in the human gut has gained increasing interest due to the broader availability of data and functional databases and substantial progress in data analysis methods, but also due to the high relevance of the microbiome in human health and disease. While most analyses infer interactions among highly abundant species, the large number of low-abundance species has received less attention. Here we present a novel analysis method based on Boolean operations applied to microbial co-occurrence patterns. We calibrate our approach with simulated data based on a dynamical Boolean network model from which we interpret the statistics of attractor states as a theoretical proxy for microbiome composition. We show that for given fractions of synergistic and competitive interactions in the model our Boolean abundance analysis can reliably detect these interactions. Analyzing a novel data set of 822 microbiome compositions of the human gut, we find a large number of highly significant synergistic interactions among these low-abundance species, forming a connected network, and a few isolated competitive interactions.

## Author summary

Over the last years the composition of microbial communities in the human gut, the gut *microbiome*, has gained prominence in clinical research. Providing an estimate of the microbial interaction network from compositional data is an important prerequisite for clinical interpretation and for a better theoretical understanding of such microbial communities. Many studies have focused on the dominant interactions of species that are highly abundant such as, on the phyla level, *Bacteriodetes* and *Firmicutes*. Using binarized abundance vectors (recording only the presence and absence of microbial species) we show that the low-abundance segment of the microbiome also contains a large number of systematic interactions. For low-abundant species, our inference method evaluates the transformation of pairs of such vectors ‘binary co-abundance’ under Boolean operations. First we calibrate our new method using simulated data. Then we apply it to novel microbiome data from a human population study. The method reveals a large number of significant positive interactions and several significant negative interactions among low-abundance microbial species. It can be argued that important inter-individual differences and adaptations to changes in environmental conditions rather occur on the level of the low-abundance species than in the few main highly abundant species. This hypothesis could explain the broad distribution of abundances in microbiome compositions.

## Introduction

An important current trend in the analysis of microbiome compositions is to relate co-abundance patterns with functional capabilities of the microbial species [1–3]. Examples of such analyses include the use of phylogenetic relationships as a proxy for functional similarity [4], the statistical analysis of an overlap in enzyme content [5], up to the study of metabolic networks of interacting species via the definition of environmental boundaries of metabolic networks [6] to the concept of metabolic interactions between microbial species [7]. Network approaches are an important ingredient in this endeavor of relating ecological and functional aspects of microbiome composition [8, 9]. Mathematical models of the microbiome system that compare to data still are rare or address specific situations. For instance, [10] modeled the primary succession of murine intestinal microbiota. This is a case where relatively well-defined initial conditions are available such that theory-experiment comparison becomes feasible. Also concepts from ecological community theory seem promising in explaining microbiome composition. [11] review approaches where environmental selection, habitat types, and invasion processes after disturbance lead to different scenarios of community assembly. But in general, a “theory of the microbiome”, especially with the ambition to provide clinical relevance, is far from being established.

In addition to the obvious challenge of finding a suitable representation of functional capabilities, it is also not clear, how co-abundance patterns reliably reveal the set of (synergistic and competitive) microbial interactions. Network effects (i.e. the multitude of positive and negative influences acting upon each microbial population and affecting the abundance pattern) will impede the link-by-link inference of such microbial interactions. Using simulated abundance patterns and analyzing a large number of stool microbiome samples from a community-based sample, we address the statistical question, how reliably co-abundance patterns reveal (the set of synergistic and competitive) microbial interactions.

Recently there has been evidence for few discrete stable compositional states, or enterotypes [12]. While this viewpoint has been challenged in the last years [13, 14], the hypothesis that microbiome compositions may follow a few distinct patterns remains actively discussed in research. It is especially not clear in general whether microbiome abundances are comprised of clustered states (enterotypes) or merely assume different values in a “gradient”-like landscape [13].

Over the last years the potential relevance of the human microbiome for aspects of health and diseases have become ever more apparent [2]. Along with this development goes an increased interest from theoretical biology and systems biology to understand the microbiome, its stability, its contributions to disease onset and progression, as well as its response patterns to perturbations.

These debates, together with the availability of ever more data sets on microbiome compositions, emphasize the importance of a theoretical understanding of the dynamical properties of the microbiome. In this context, the inference of microbial association networks from species abundance data has a very important role [8]. Commonly applied methods are regression analysis, local similarity analysis and statistical validations via suitable null models (see [8] for a detailed review on network inference methods in the context of microbiome compositions). In [9] a data analysis pipeline with several correlation and similarity measures as well as *Generalized Boosted Linear Models* have been used to reconstruct microbial interaction networks across different body sites based on the Human Microbiome Consortium data.

In general the inference of the underlying interaction network is a nontrivial task. To address side effects of normalization and statistically under-powered data, [15] introduced a new transformation and graphical inference framework and demonstrated improved detectability of the interaction network for a suitably selected sparsity parameter.

A striking feature of microbiome compositions is the wide spread of abundances: Although often dominated by a few highly abundant species, a typical microbiome in the human gut also consists of a wide range of low-abundance species. It seems plausible that the main ‘housekeeping functions’ of such a system of interacting microorganisms are installed by the major, high-abundance components, while subtle adjustments to environmental changes and differences in the host phenotype are achieved rather via this broad range of low-abundance species (see e.g., [16, 17]). However their detection is more challenging, as network reconstruction methods based on abundance tend to priorize interactions involving the high-abundance species.

In this work, we present and analyze a comparatively large dataset (consisting of 822 samples) and introduce a new network inference method based on Boolean networks. The purpose of our investigation is two-fold: First, we introduce a new method for inferring microbial interaction networks from abundance data and test this method using simulated ‘data’. Second, we apply this method to a new data set of human gut microbiome compositions and show that the co-abundance patterns among low-abundance species contains a multitude of highly systematic, statistically significant interactions.

The microbial interaction networks obtained from most analyses are dominated by highly abundant species. Here the negative interaction between *Firmicutes* and *Bacteriodetes* is a prominent example, which is also a main basis of the concept of enterotypes. As discussed above, here we assess, whether the low-abundance segment of the microbiome contains evidence for systematic interactions. Therefore, throughout this study we focus on binary data (i.e. the presence or absence of a microbial species in a particular microbiome sample). A simple example illustrates the statistical signal we focus on in these binarized vectors: On a link-by-link basis (i.e., for a single pair of microbial species), a preference for (1, 1) in positive interactions or (1, 0) and (0, 1) in negative interactions can be expected. It is not clear, however, whether this tendency is also visible in a whole network, where nodes tend to have more than one such positive or negative interaction.

Lastly, it is an open question, whether ‘snapshots’ of steady states of the system (instead of, e.g., time courses) allow a reliable reconstruction of such interactions.

The main difference of our approach to previous studies is that we binarize the data and make use of the full methodological scope available on this binary state space. This is seen (i) in the simulation model we employ to calibrate and test our method (section *Method for testing the analysis method using simulated data*), (ii) in the computational technique we use to distinguish between cooperative and competitive interactions (sections *Boolean abundance analysis* and *Analysis of simulated data*) as well as in (iii) the possibility to treat some of the properties of the binarized abundance vectors analytically (section *Background*).

For the simulation model, we construct random graphs of positive and negative interactions, simulate time courses starting from random initial abundance patterns and thus obtain a set of attractors (i.e. binary steady state vectors), which represent steady-state microbiome compositions on a binarized (species is present or absent) level, arising in a network of cooperative (synergistic) and competitive (antagonistic) interactions. In general, Boolean network models [18] have been very successful in the context of gene regulation: Ignoring the gradual changes of gene activity and rather focusing on the logical organization of the system, this logical circuitry determines the patterns of ‘on’ and ‘off’ states of genes [19]. The binary nature of our data allows us to use the change of co-occurence vectors under Boolean operations as a predictor of the sign of the interaction. A decisive test is, how our new measure performs under noise and with increasing connectivity.

## Methods

### Boolean abundance analysis

Here we introduce a new method for the inference of microbial interaction networks from microbiome composition data. The method, called Entropy Shifts of Abundance Vectors under Boolean Operations (ESABO), evaluates the information content of pairs of binary abundance vectors, when combined via Boolean operations. In contrast to purely descriptive abundance diversity measures used in population ecology, here we introduce an approach which is directly targeted for the detection of (synergistic and competitive) relationships among microbial species. The ESABO method starts from a set {*k*} (1 ≤ *k* ≤ *N*_*A*_) of samples with abundances $b_{k}^{\left(i\right)}$ for each species *i*. Let OP be a Boolean operation (OP ∈ {AND, OR, NAND, NOR, …}). Then the ESABO score for operation OP of species *i* with respect to species *j* is defined as the z-score (compared to a null model of shuffled abundance vectors) of the entropy of abundances after pointwise application of a Boolean operation

$$\begin{array}{c} {\left(x_{ij}^{i\;\text{OP}\;j}\right)}_{k}⟵b_{k}^{\left(i\right)}\text{OP}\;b_{k}^{\left(j\right)} \end{array}$$ (1)

where the z-score is obtained from comparison to an ensemble of

$$\begin{array}{c} {\left({\tilde{x}}_{ij}^{i\;\text{OP}\;j}\right)}_{k}⟵b_{k}^{\left(i\right)}\text{OP}\;{\tilde{b}}_{k}^{\left(j\right)} \end{array}$$ (2)

where ${\tilde{b}}_{k}^{\left(j\right)}$ for each fixed *j* is an abundance vector randomly reshuffled in *k*. The entropy *H* = −∑_*i*_ *p*_*i*_ ln *p*_*i*_ (in physics with prefactor *k*_B_ or in Shannon theory of information with base 2) of any normalized set of probabilities *p*_*i*_ is a measure of uncertainty. Here, the sum is over the two states 0 and 1 occurring in the vector ${\left(x_{ij}^{i\;\text{OP}\;j}\right)}_{k}$. An entropy shift therefore can be associated with a gain of information. In it is shown that the occurring patterns can be extracted from one operation and we choose the AND operation which appears more straightforward to interpret.

### Method for testing the analysis method using simulated data

The ESABO method only uses species occurrences as a binary information. In order to calibrate and test our analysis method, we therefore opted for a minimal model, which creates such occurrence patterns on a binary level.

We generate a random undirected graph with *N* nodes, representing *N* microbial species, connected by *M*_+_ positive and *M*_−_ negative interactions. Starting from random (binary) compositions, we update the state of the system according to the following update rule:

$$s_{i}\left(t+1\right)=\{\begin{array}{rr} 1, & \sum_{j=1}^{N}G_{ij}s_{j}\left(t\right)>0\\ s_{i}\left(t\right), & \sum_{j=1}^{N}G_{ij}s_{j}\left(t\right)=0\\ 0, & \sum_{j=1}^{N}G_{ij}s_{j}\left(t\right)<0 \end{array}$$ (3)

where *G* = (*G*_*ij*_) is the generalized adjacency matrix of the interaction graph *G*: *G*_*ij*_ = 1, −1, 0 for positive, negative or no interactions between species *i* and *j*, respectively. For each species *i* at time *t*, *s*_*i*_(*t*) specifies whether the species is abundant (*s*_*i*_(*t*) = 1) or absent (non-abundant, *s*_*i*_(*t*) = 0).

From the networks we go via simulated time series across 1000 random initial conditions to asymptotic compositions. In most cases, the observed attractor is a steady state (see Supporting Information in S1 Text for details). In cases where a cyclic attractor is observed, the recorded asymptotic composition will be one of the time points from the cycle. For each interaction network *G*, we thus obtain a list of attractor vectors ${\vec{a}}^{\left(j\right)}$ with *j* = 1, …, *N*_*A*_, where *N*_*A*_ denotes the number of (numerically observed) attractors. Each such vector can be seen as an experimental sample of microbial abundancies (preserving only the information, whether a species is present or absent).

**Fig 1:**
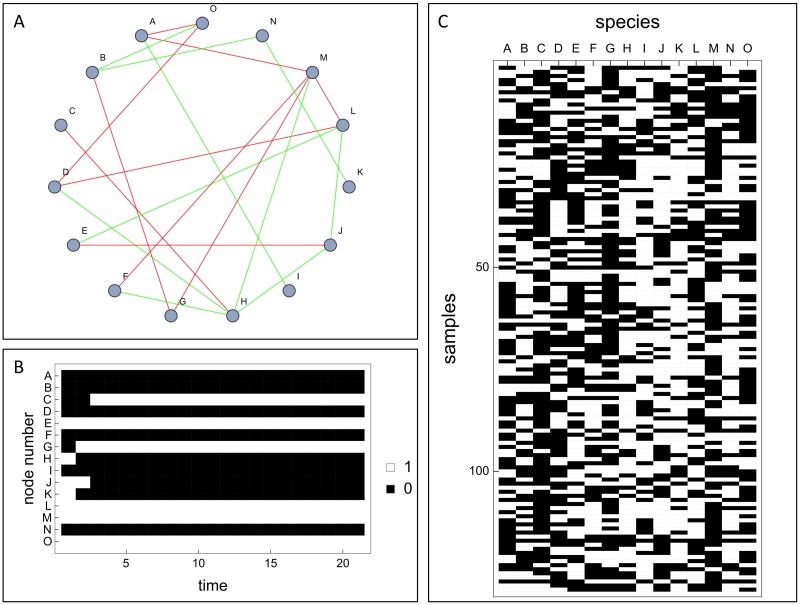
(A) Example of a species interaction network (*N* = 15, *M*_+_ = *M*_−_ = 10) used to generate synthetic data of microbial abundances. The positive (negative) links are displayed in green (red) colour or respectively light gray (dark grey). (B) Time course obtained from recursively updating a random abundance pattern for the species interaction network from (A) according to the update rule, Eq (3). (C) Data matrix *A*_*ij*_ showing all (*N*_*A*_ = 129) numerically observed attractors for the network from (A).

Such a row vector ${\vec{a}}^{\left(j\right)}$ of the data matrix $A_{ij}=a_{i}^{\left(j\right)}$ is in the following called the *occurrence vector* of the *j*th sample. The column vector ${\vec{b}}^{\left(i\right)}=\left\{a_{i}^{\left(1\right)},a_{i}^{\left(2\right)},\ldots ,a_{i}^{\left(N_{A}\right)}\right\}$ are named the *abundance vector* of the *i*th species across all *N*_*A*_ samples. Fig 1 illustrates this setup.

In the following, we analyze co-abundances, i.e. the relative frequencies (approximating pair probabilities) $p_{kl}^{\left(i,j\right)}$ of entries (*k*, *l*) in pairs of abundance vectors $\left({\vec{b}}^{\left(i\right)},{\vec{b}}^{\left(j\right)}\right)$ with *k*, *l* ∈ {0, 1}. Furthermore, let $p_{k}^{\left(i\right)}$ denote the relative frequency of the entry *k* in the abundance vector ${\vec{b}}^{\left(i\right)}$. Then the Jaccard index with respect to (1, 1) is defined as $J_{11}\left(i,j\right)=p_{11}^{\left(i,j\right)}/\min\left(p_{1}^{\left(i\right)},p_{1}^{\left(j\right)}\right)$.

We analyze pairs of abundance vectors via their transformation under Boolean operations. Let $x_{ij}^{\left(\text{AND}\right)}$ be the binary vector obtained from applying a logical AND to the two vectors ${\vec{b}}^{\left(i\right)}$ and ${\vec{b}}^{\left(j\right)}$, i.e., ${\left(x_{ij}^{\left(\text{AND}\right)}\right)}_{k}=b_{k}^{\left(i\right)}\text{AND}\;b_{k}^{\left(j\right)}$, leading to relative frequencies $p_{0}\left(x_{ij}^{\left(\text{AND}\right)}\right)$ and $p_{1}\left(x_{ij}^{\left(\text{AND}\right)}\right)$ of zeros and ones in the resulting vector (which is of length *k*).

The entropy $H^{\left(\text{AND}\right)}\left({\vec{b}}^{\left(i\right)},{\vec{b}}^{\left(j\right)}\right)=-\sum_{k}p_{k}\left(x_{ij}^{\left(\text{AND}\right)}\right)\log p_{k}\left(x_{ij}^{\left(\text{AND}\right)}\right)$ is then an indicator, whether the vector has become simpler or less simple under the Boolean operation. This ‘entropy shift’ is the main observable in the ESABO method introduced in section Boolean abundance analysis. These entropies can now be compared with entropies obtained from shuffled versions of the original abundance vectors ${\vec{b}}^{\left(i\right)}$ and ${\vec{b}}^{\left(j\right)}$, leading to a z-score (of entropies compared to the entropies from the shuffled versions). This comprises the ESABO score for species pair (*i*, *j*), which can be expected to be markedly different for positive and negative interactions between species *i* and *j*. In Table 1 the ESABO scores for the network from Fig 1A are shown. The z-scores were always calculated with respect to 1000 randomized networks. Except for one outlier in the case of synergistic links, our method successfully classifies the respective signs of interaction links with high significance |*z*—*score*| ≫ 1.

**Table 1:** Discrimination between synergistic and competitive links on the level of the z-scores for the entropy shifts under a logical AND. Values are given for the ten positive and ten negative interactions of the network from Fig 1A.

Synergistic links		Competitive links	
H •-• M	3.58356	A •-• O	−5.03179
E •-• L	2.69176	D •-• O	−4.84954
K •-• N	−55.875	D •-• L	−7.11475
D •-• H	4.2044	F •-• M	−2.18863
A •-• I	4.59704	E •-• J	−5.52525
J •-• L	2.90282	G •-• M	−5.6133
B •-• O	4.43435	B •-• G	−13.1663
H •-• J	3.16823	C •-• H	−15.8969
B •-• N	1.6011	A •-• M	−6.42405
F •-• H	3.41507	L •-• M	−6.70737

Simulating ‘abundance data’ already in a binarized form allows us to study interaction patterns not masked by the extreme hierarchy of species abundances. However, the Boolean model here only serves the purpose of testing and calibrating the method. It is by no means intended to produce ‘data’ which are in all aspects similar to the true microbial abundance data. In particular, in this minimal model the number of attractors decreases rapidly with the number of links (see Fig D in the Supporting Information S1 Text).

### Study subjects and sample collection

822 individuals from a community-based sample from Schleswig-Holstein (Germany) were used as *discovery sample set*. The stool samples, as well as corresponding phenotypic data and information on diet and nutrition were collected by the PopGen Biobank (Schleswig-Holstein, Germany) [20]. Study participants collected fecal samples at home in standard fecal tubes. Samples were shipped immediately at room temperature to the PopGen laboratory. Upon arrival into study center (within 24 hours) samples were stored at −80°C until processing. Studies exploring the impact of storage conditions on the samples quality and stability of the microbial communities indicated that storage in RT for 24 hour is recommended for optimal preservation [21, 22]. Written, informed consent was obtained from all study participants and all protocols were approved by the institutional ethical review committee in adherence with the Declaration of Helsinki Principles. 16S rRNA sequencing, genotype, nutritional, and phenotype data used for the herein described study has been made available to other scientists through PopGen’s biobank general data transfer agreement.

### Genotyping data—Verification of gender and ancestry

Dense single nucleotide polymorphisms (SNP) genotype data set (n = 1,074,163 SNPs) derived by combining and quality controlling—using standard methods of data filtering—from Affymetrix 6.0, Affymetrix Axiom arrays and the custom Illumina Immunochip and Illumina Metabochip was used for verification of gender and ancestry of study individuals. Individuals who showed statistically relevant genetic dissimilarity to the other subjects (population outliers identified by PCA-based mapping against the HapMap III CEU, CHB, JPT and YRI population) or who showed evidence for cryptic relatedness to other study participants (unexpected duplicates, first- or second-degree relatives identified by identity by descent estimated using the R-package SNPRelate (vs. 0.9.19)) were removed. All gender assignments could be verified by reference to the proportion of heterozygous SNPs on the X chromosome. The final data set consisted of 784 samples.

### Isolation of fecal DNA and multiplex sequencing

The bacterial genomic DNA for the discovery sample set was extracted manually using MoBio PowerSoil DNA Isolation Kit. The discovery sample set was sequenced using primers amplifying V1-V2 regions of 16S rRNA gene combined with Multiplex IDentifiers (MIDs) and adapters established for the a 454 Life Sciences GS-FLX using Titanium sequencing technique as described in [23].

### Microbiome data analysis

Quality filtering of the 454 GS-FLX data was performed according to [24] in summary only reads that are at least 250 bp long and average quality >25 were kept. The microbiome of *discovery sample set* was subsetted to 1000 reads per sample and taxonomical census matrix from phylum to genus level were constructed accordingly. Phylogeny based alpha-diversities (Faith PD) and beta-diversities (weighted and unweighted Unifrac) were calculated with FastTree produced maximum-likelihood tree and Mothur.

## Results

### Analysis of simulated data

**Fig 2:**
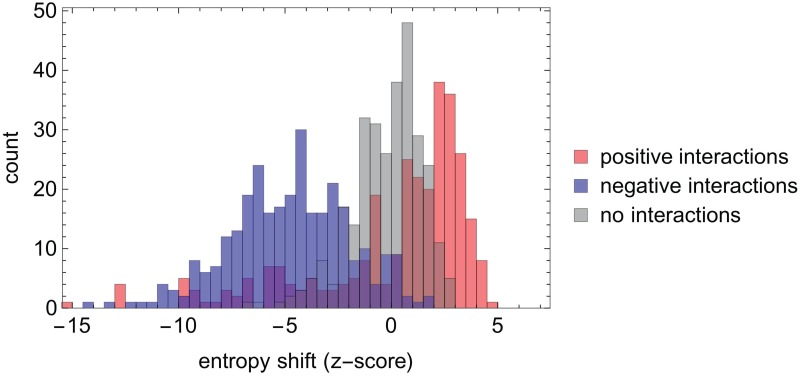
Histogram of z-scores of entropy shifts obtained with the ESABO method (Boolean AND) applied to simulated binary abundance patterns from 20 species interaction networks consisting of 15 nodes and 15 positive and negative interactions, respectively. Blue: negative interactions, red: positive interactions, gray: random sample of absent links. (Note that ‘mixed colors’ appear, when histograms overlap.)

First, we test the ESABO method using simulated data, as discussed in Section *Boolean abundance analysis*. In order to better understand the prediction quality of the ESABO method within this framework of the simulated species interaction networks we evaluated the z-scores of entropy shifts under a Boolean AND for an ensemble of 20 networks (*N* = 15, *M*_+_ = *M*_−_ = 15) for positive interactions, negative interactions and a random selection of absent interactions (see Fig 2). The histogram for negative interactions is clearly centered at negative z-scores, while the positive interactions are predominantly in the positive range, even though some values are in the negative z-score range as well. These outliers will be discussed in more detail below. The sample of absent links yields a narrow distribution of z-scores around zero, confirming that we can expect only a small contribution from false positives in the ESABO method.

In the subsequent analysis, we will condense the information contained in the ESABO score even further and define the prediction quality in the following way (see also Supporting Information): The prediction quality of positive interactions is the number of times a z-score larger than 1 is observed minus the number of times a z-score smaller than −1 is found, divided by the number of positive interactions. For negative interactions, negative z-scores are expected. Correspondingly, the prediction quality is the number of times a z-score smaller than −1 is observed minus the number of times a z-score larger than 1 is found, divided by the number of negative interactions. In the case of the Jaccard index *J*, the prediction quality is the number of times with *J* > 0.6 minus the number of cases *J* is greater than 0.4 minus the number of cases *J* is smaller than −0.4.

The range of connectivity values is limited by two requirements: (1) We only consider connected networks. (2) We require more than 100 distinct steady states. Furthermore, we analyze networks with the same number of positive and negative interactions (*M*_+_ = *M*_−_).

**Fig 3:**
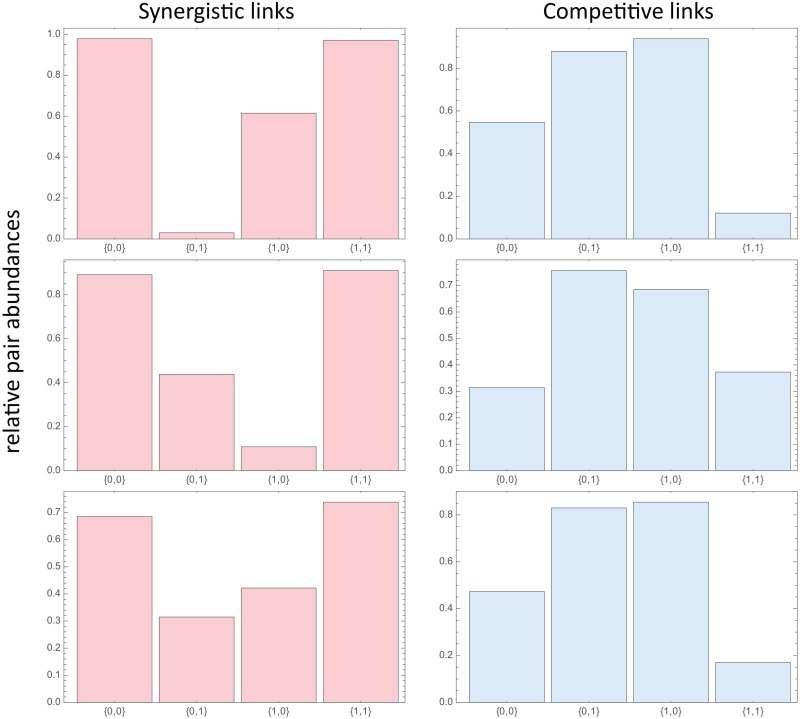
Examples of (normalized) pair probabilities $p_{kl}^{\left(i,j\right)}/\min\left(p_{k}^{\left(i\right)},p_{l}^{\left(j\right)}\right)$ with *k*, *l* ∈ {0, 1} for six links of the network from Fig 1A: Three positive interactions (lhs) and three negative interactions (rhs).

Even on the level of the pair probabilities $p_{kl}^{\left(i,j\right)}$, the difference between positive and negative interactions is clearly seen. Fig 3 shows some examples of histograms of the corresponding relative pair abundances, $p_{kl}^{\left(i,j\right)}/\min\left(p_{k}^{\left(i\right)},p_{l}^{\left(j\right)}\right)$ for the small example from Fig 1A. This systematic difference of positive and negative interactions derived from a large set of *steady state* composition is a key result of our investigation.

The standard Jaccard index, for example, would pick up a systematic enhancement (suppression) of the co-occurences of 1’s (i.e. the pair (1, 1)) for positive (negative) interactions. It is our hypothesis that the amount of change (amount of simplification) two vectors display under a Boolean operation (e.g., logical AND or logical OR) is very different for synergistic and competitive interactions. In addition, this systematic change is quite robust against ‘cross talk’ generated by additional links and against ‘noise’ generated by measurement errors in the data.

In the following, we will use the simulated data to investigate the prediction quality of such entropy shifts under increasing connectivity and noise, and benchmark it against the Jaccard index, which is a more standard analysis method of species co-abundances.

**Fig 4:**
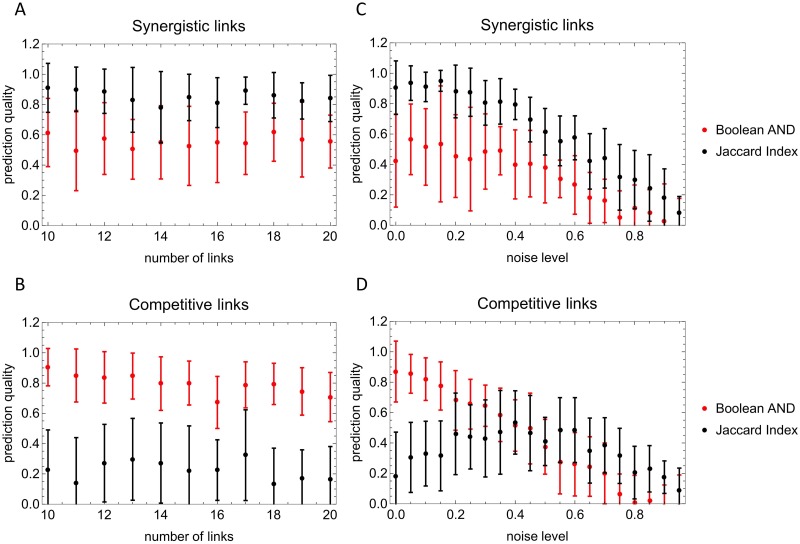
Prediction qualities as a function of the number of links *M*_+_ = *M*_−_: (A: Positive interactions) and (B: Negative interactions) and as a function of the noise level in the data: (C: Positive interactions) and (D: Negative interactions). Parameters used: network size *N* = 15, averages have been performed over 20 networks, 200 randomizations have been performed for the z-score computation; for the noise level dependence, *M*_+_ = *M*_−_ = 10.

We find that the entropy shift performs less well than the Jaccard index in identifying positive interactions, but substantially better in indentifying negative interactions (Fig 4). Both measures are similarly robust with respect to connectivity and random entries in the data (noise). The interesting observation of a maximal prediction quality of the Jaccard index at intermediate noise levels (Fig 4D) might call for additional investigations.

The Jaccard index is here used on the binary level as follows. For positive links, the frequency of (1, 1) in two binarized vectors is normalized by the minimum number of 1s in each vector. For negative links, the frequency of (0, 0) in two binarized vectors is normalized by the minimum number of 0s in each vector. The comparison with the Jaccard index only serves the purpose of showing that our assessment based on the entropy shift achieves a similar quality. The prediction quality here is defined as (normalized) number of correctly classified links minus the number of incorrectly classified links. A prediction quality of 0.5 thus means that 50 percent *more* links are correctly classified than incorrectly classified.

The ESABO method is about the statistics of pairs of binary values. The main variant is the one, where entropy shifts under Boolean operations are evaluated. In Fig 4 this standard version is compared with a variant of the Jaccard index applied to the binary vectors (see the Supporting Information S1 Text for the detailed definition). In spite of the high prediction quality obtained with the Jaccard index, the disadvantage of the ESABO version using the Jaccard index is that the thresholds for determining a positive or negative interactions are somewhat arbitrary, while in the original ESABO score (i.e., the z-score of entropy shifts under a Boolean AND) the threshold has a clear interpretation as the number of standard deviations away from random data. In subsequent versions of ESABO we will study particularly, how *combinations* of Boolean operations and such simple indices can be employed to enhance prediction quality further.

It is important to sample the system’s dynamical ‘possibility space’ (i.e., the set of steady states) homogeneously. We found that a sampling according to the system’s attractor basin sizes systematically reduces the detectability of edges (see Fig. C in S1 Text).

In order to verify that the entropy shifts evaluated within the ESABO method are robust against a certain amount of randomness (detection errors in microbial species), we introduce binary noise in the simulated data. A noise level *p* means that *p* percent of entries in a binarized abundance vector are substituted by a random choice of 0 and 1. We observe that the prediction quality remains rather high up to noise levels of 20 percent (*p* = 0.2; see Fig 4).

With the inclusion of simple binary noise we can verify that the reconstructed links are robust against detection errors in the data, an issue that can be expected to be of much higher relevance in the case of low-abundance species than in the high-abundance regime.

As seen in Table 1 and Fig 2 there are occasional ‘outliers’ in the z-score distributions (positive interactions with a large negative z-score). We have performed several analyses to understand, whether these outliers can be predicted from the topology of the species interaction network. So far, we have not found a topological explanation for this effect. Based on 40 random species interaction networks (*N* = 15, *M*_+_ = *M*_−_ = 15) and 500 runs on each of the networks we estimate the number of such outliers (z-score ≤ − 1 to be around 9.6 percent of all positive interactions (see Supporting Information S1 Text for details). We have observed that the outliers are associated with strong compositional differences between the two binarized vectors entering the ESABO score. This point will be investigated in more detail in the future.

### Analysis of the human gut microbiome compositions

In the previous section we have shown that the abundances and co-abundances stemming from positive and negative interactions can be detected from ESABO scores of the dynamically generated attractor states. To apply this to biological abundance data, we analyze the co-occurences on phyla level for the dataset described in subsection *Study subjects and sample collection* and the subsequent three *Methods* subsections. A binarization threshold of 1 has been used (i.e. values of zero are mapped to zero, while all other values are mapped to one), as the distinction between the presence and absence of a species seems quite reliable (see Supporting Information S1 Text).

We observe that the pairs with highest (and lowest) ESABO scores are strongly symmetric, i.e., we observe positive mutualisms or antagonims, respectively. We here compute the ESABO scores with respect to logical AND operations. A large number of z-scores is observed in the range of absolute values between −1 and 1.5.

We note that the overall resource competition is expected to lead to a more ubiquitious-type connectivity (i.e., highly clustered or even close to all-to-all coupled within the subgraph) such that only highest z-scores are considered here. Correspondingly, the threshold for positive co-occurence has to be adjusted independently. In total, we obtain 4 competitive (ESABO score) resp. 8 lowly co-abundant (by z-score) pairs of nodes as listed in Table F and C, and a fairly more extensive list of mutualistic (and highly co-abundant) pairs of phyla shown in Tables D+E and F. in the Supporting Information S1 Text.

### Co-abundance networks: Positive and negative interactions

**Fig 5:**
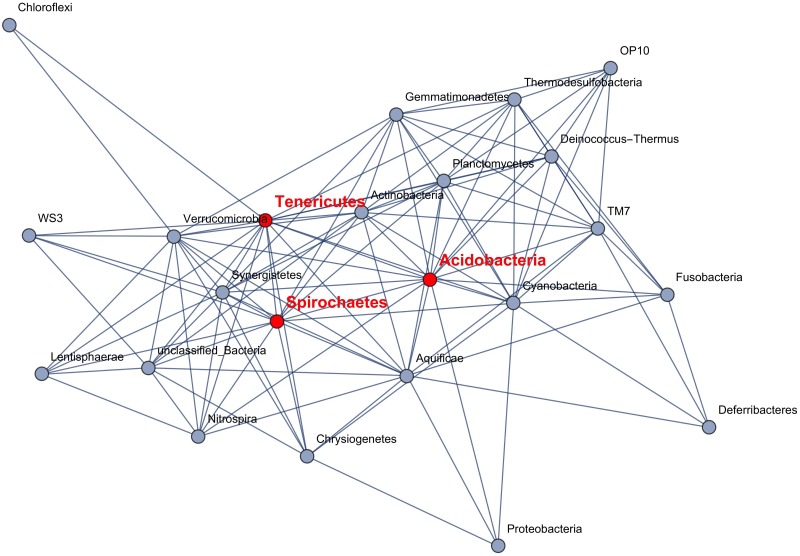
Network of mutualistic interactions: Pairs of microbes where the Boolean operation AND leads to a high entropy shift (compared to 1000 randomizations). Only links with an ESABO score ≥ 1.0 are shown. The edges shown can be interpreted as cooperative mutualistic relationships. Nodes referred to in Fig 7 are highlighted in red.

**Fig 6:**
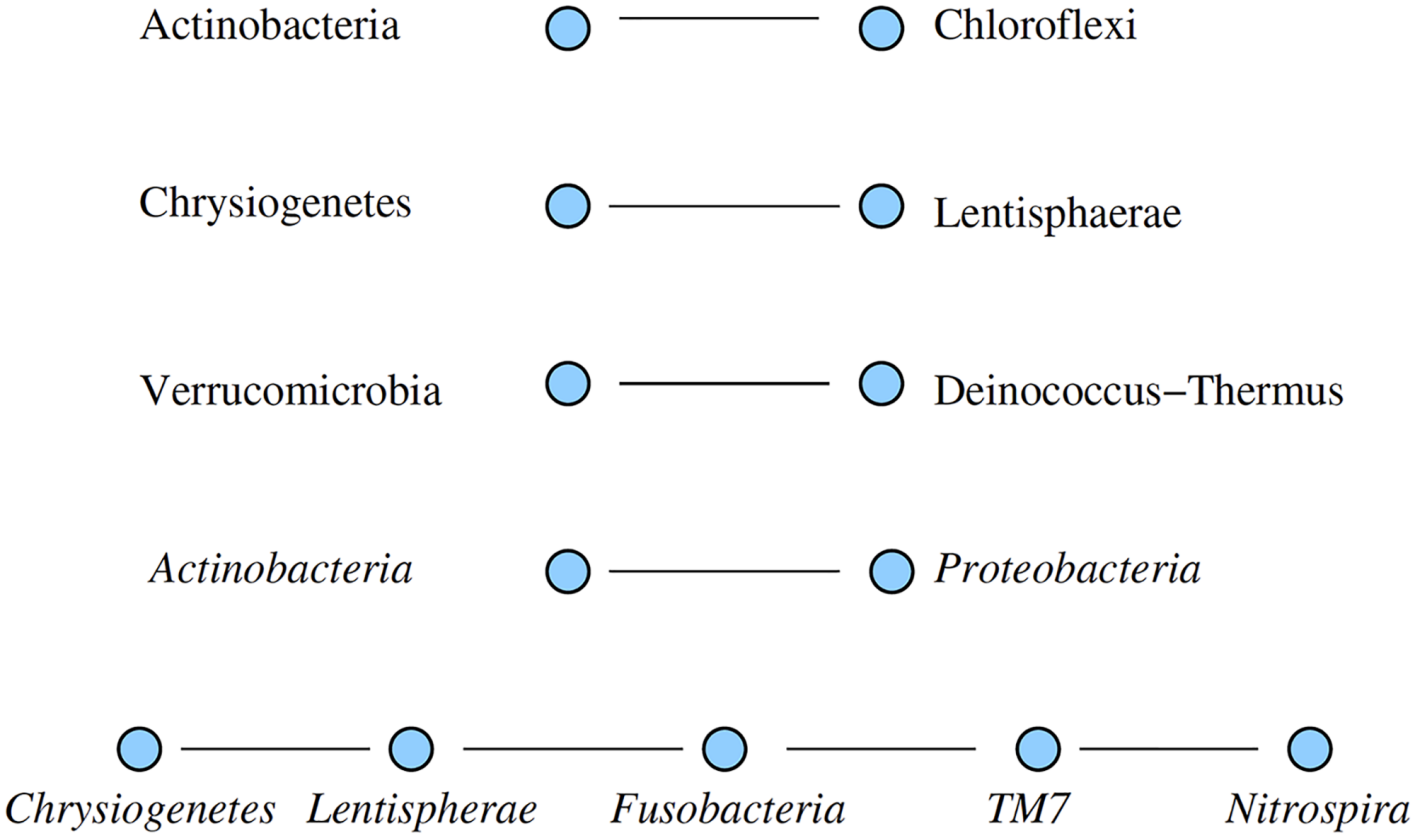
Network of pairs of microbes (phyla) where the co-occurence is less compared to random pairing. The *Actinobacteria*—*Proteobacteria* link is only detected by the entropy shift (ESABO score ≤ − 1), the five-phyla chain is only detected by the co-occurence analysis. The first three links have high z-score values for both methods. As these links are to be interpreted as competition between the species, each subgraph describes a network of mutually suppressing microbes.

From the co-abundance data and their respective ESABO scores we can extract a network of significantly mutualistic links between species (Fig 5) and a corresponding network of mutually inhibiting links (Fig 6).

Interestingly, the competitive and cooperative links form different networks. For competition and thus low co-occurence, the nodes are fragmented into 4 subgraphs (see Fig 6). For mutualism and thus high co-occurence, the nodes form a connected graph which contains *Tenericutes*, *Actinobacteria*, and *Spirochaetes* as the three nodes with highest node degree (Fig 5).

**Fig 7:**
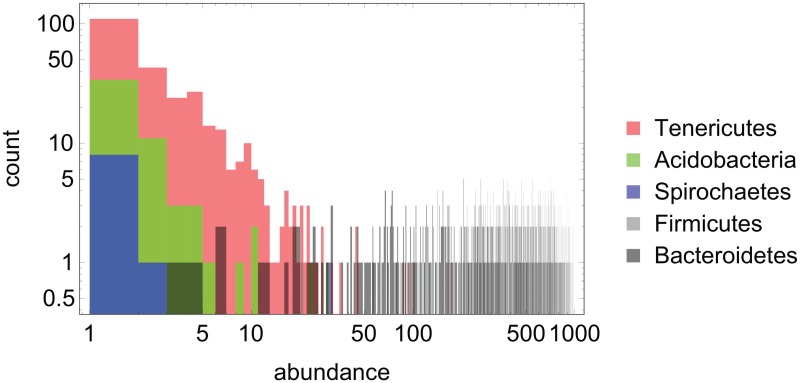
Histogram of abundance values (extracted from the 822 microbiome compositions; see Methods) for the two high-abundance phyla, *Bacteroidetes* and *Firmicutes*, as well as the three phyla with the highest degree *k*_+_ in the network of positive interactions shown in Fig 4, namely *Tenericutes* (*k*_+_ = 16), *Acidobacteria* (*k*_+_ = 16) and *Spirochaetes* (*k*_+_ = 14). The abscissa displays the count number (of samples) in which a relative abundance (1…1000) is observed for the respective phylum.

The histogram of abundances for the three main hubs in the network of positive interactions, Fig 7, illustrates that our method is sensitive to interactions among low-abundance species. As a contrast, the abundance histograms for the two dominant phyla, *Bacteroidetes* and *Firmicutes* are also shown.

## Discussion

Human microbiome compositions are currently of high interest, especially in clinical contexts as an important data resource for the characterization of clinical phenotypes and as a source of potential biomarkers for disease progression and treatment response. Specifically, in clinical research, the gut microbiome has been linked to several disease conditions [25]. Therefore, understanding the determinants and compositions of the gut microbiome in health and disease is of high importance. To understand natural microbiome compositions, sufficiently large population studies (non-clinical controls) have to be analyzed. Here we presented data on 822 microbiome compositions from a community-based sample, together with a novel analysis method, called ESABO, based on the entropy shift of pairs of—binarized—abundance vectors under Boolean operations.

We have calibrated our framework in a natural way by a Boolean network dynamics for which the time development is determined, and leads for each possible initial state (microbial composition) to attractor states which can be interpreted as estimates for the microbe density patterns that are expected in a population of humanindividuals. In the biological context, this is the relevant picture because a low-abundant species, due to a new nutrient or to disease-related metabolic change, can grow into its niche and its population density adapts. Therefore on the longer timescale it is only relevant whether a microbial species is present or not, and its precise numerical value of abundance at some time point may be less important on its own.

When applied to simulated ‘data’, the method shows a convincing performance in network inference. However, the interaction patterns derived with this novel method show substantial differences to previously published results. In several studies, including the present one, the analysis and interpretation of microbiome compositions revealed different pictures so that it is still not clear which abstracted and functional structure would comprise a healthy human gut microbiome.

As suggested by [16, 17], metabolic functions may—to a nonneglible extent—be performed by low-abundant microbes. Indeed the omnipresence of a few high-abundant microbes may reflect their task of processing main nutrients and substances present in the gut, whereas several more specific functions can be on the shoulders of several low-abundant species that are specified on their “ecological niche” of processing certain metabolites, and their absence (or, in other cases, presence) therefore is expected to facilitate or accompany certain diseases or body dysfunctions.

From this viewpoint, the low-abundant species deserve additional attention. The precise investigation of the low-abundant species is however a challenging task: Co-abundances between low-abundant bacterial species are inherently difficult to measure and cast into conclusions about underlying mutualistic interactions. Introducing a new inference method (SPIEC-EASI), [15] (see their Fig 6) demonstrated for the American Gut network data that connections between the phyla *Bacteriodetes* (comprised by families *Bacteriodacae*, *Porphyromonadacae*, *Rikenellacae* and bacteria from order *Bacteriodetes* with unclassified family) and *Firmicutes* (comprised by families *Lachnospiracae*, *Ruminococcacae*, *Streptococcacae*, *Erysipelotrichacae* and bacteria from order *Clostridiales* with unclassified family) are mainly inhibitory. While the authors report a high agreement of a core network made apparent by four different inference approaches, the inter-phyla links between the clusters of bacteria strongly disagree between the approaches, making it difficult to judge whether the underlying interactions are neutral or inhibitory. For the interactions between low-abundant bacteria the situation is even more difficult. As besides *Bacteriodetes* and *Firmicutes* only *Proteobacteria* (comprised by families *Enterobacteriacae* and *Pseudomonadacae*) are considered, the remaining aggregated network of phyla would contain only one node such that we cannot compare our low-abundance analysis directly. The study by Kurtz et al.—comparing the result of four different inference schemes—however clearly confirms a large connectivity by positive interactions within the phyla.

It is interesting to compare our results to the co-occurence analysis by [9]. When restricting the data underlying Fig 4 therein to the abundances in the gut, aggregating links on the phylum level (see Table H in S1 Text) then it is not astonishing that the competitive link between *Bacteriodetes* (4) and *Firmicutes* (11) results from a count of −116. All other links are, in comparison to this strongest one, weak (between −9 and +4) and are displayed in Fig F and listed in Table H in the Supporting Information S1 Text. However, if *Bacteriodetes* and *Firmicutes* are removed from this network, the whole network reduces to

Proteobeacteria (18)⇔Unclassified (26)⇔Lentispherae (14)

where both mutualistic links are weakly positive (+2 each). The interpretation of this network is weakened by the fact that both links connect to unclassified phyla such that a clear microbiological interpretation is not immediate. Subsequently, this re-analysis of the dataset reveals no interaction links on the phyla level, whereas the ESABO analysis on our dataset is able to highlight several positive as well as negative links.

Summarizing, when applied to simulated ‘data’, our ESABO method shows a convincing performance in network inference. However, the interaction patterns derived with this novel method show substantial differences to previously published results. In particular, the recent study of co-occurrences between and within different body areas derived from the Human Microbiome Consortium data [9] comes to a markedly different interaction network.

Marino et al. observed far more negative than positive interactions [10]. In contrast, we identify only a handful of negative links, and a large body of positive links.

While our raw data confirms the negative correlation between *Bacterioidetes* and *Firmicutes*, (as reported in [9] and other work), here we look at all interactions with a method designed (due to the binarization) to focus on low-abundance species.

It should be noted that when looking at such microbial interactions on a microscopic level, the situation is more involved than a single interaction network can reveal. We might for example expect synergies between some metabolic functions, even in cases of a generally competitive interaction.

It is, of course, well known that different similarity measures can lead to markedly different inferred networks (see, e.g., the detailed discussion in [8]). Binarized abundance vectors can be sensitive to different interaction types than detected via methods focusing on gradual abundance differences. The expectation of an antagonistic interaction of *Bacteriodetes* and *Firmicutes* is for example mostly due to the observation of *changes* in microbiome composition (see, e.g., [26]).

Exploring the quality of network inference and interaction prediction using abundance ‘data’ simulated with time-continuous predator-prey models is a natural next step for our investigation. However, even the ‘data’ simulated with the Random Boolean Network (RBN) model described here allowed us to observe some interesting features of our reconstruction method:

- It was not clear beforehand, whether the sampling of asymptotic states (i.e. the RBN’s attractors) would allow a reliable reconstruction of the interaction network.
- The reconstruction quality decreases, if asymptotic states are sampled according to their basin size. When attractors with small and large basins enter the analysis in equal proportions, the reconstruction quality was best.

In [10], in line with several other data analyses, a clear picture is drawn: The systematic interactions are predominantly inhibitory. Our study suggests that these strong inhibitory links are embedded in a dense systematic network of *positive* interactions among low-abundance species.

One future extension of the ESABO approach would be to address microbiome dynamics as well. This could be achieved by estimating the network from abundance patterns and then simulating time courses, which can be compared with empirical data and interpreted both metabolically and from the perspective of Boolean models. A detailed comparison of the approach from [27] would facilitate this extension to the time domain.

In [27], the concept of Boolean networks has been applied to time series of abundance data (as opposed to our analysis, where we show that Boolean rules can also be estimated from a set of steady-state mocrobiome compositions, i.e. the *attractors* of the Boolean network). In this way, in [27] a regulatory component could be added to genome-scale metabolic models of some of the microbial species involved and a novel perspective to microbiome *dynamics* could be formulated.

Ultimately, functional microbiome models built up from genome-wide metabolic models of (the most relevant) microbial species in the microbiome, coupled by metabolite exchange and analyzed via flux-balance analysis will become the modeling standard for microbiomes. Methods for inferring microbial interation networks provide important constraints for such future models. Already today, they can allow a direct interpretation of microbiome composition and help identify systematic changes in the microbial interaction pattern during diseases or in response to treatment.

### Background

#### Methodical investigation: Dependent and independent densities

An important property of the shifted entropies is that they are not pairwise independent. The reason behind this is that they are calculated from four abundance densities for *pairs* of abundances of microbe *j*_1_ and microbe *j*_2_. Here $\alpha ≔p_{00}\cdot N_{A}≔p_{00}^{\left(i,j\right)}\cdot N_{A}=p_{00}\left(b_{k}^{\left(i\right)}=0,b_{k}^{\left(j\right)}=0\right)\cdot N_{A}$ would be the number of cases that neither *j*_1_ and *j*_2_ are present, whereas both microbes are found in *δ* = *p*_11_ · *N*_*A*_ cases. Finally *β* = *p*_01_ · *N*_*A*_ denotes cases where only microbe *j*_2_ is present, and *γ* = *p*_10_ · *N*_*A*_ denotes cases where only microbe *j*_1_ is present. The normalization can be achieved by multiplying with (*α* + *β* + *γ* + *δ*)^−1^.

**Fig 8:**
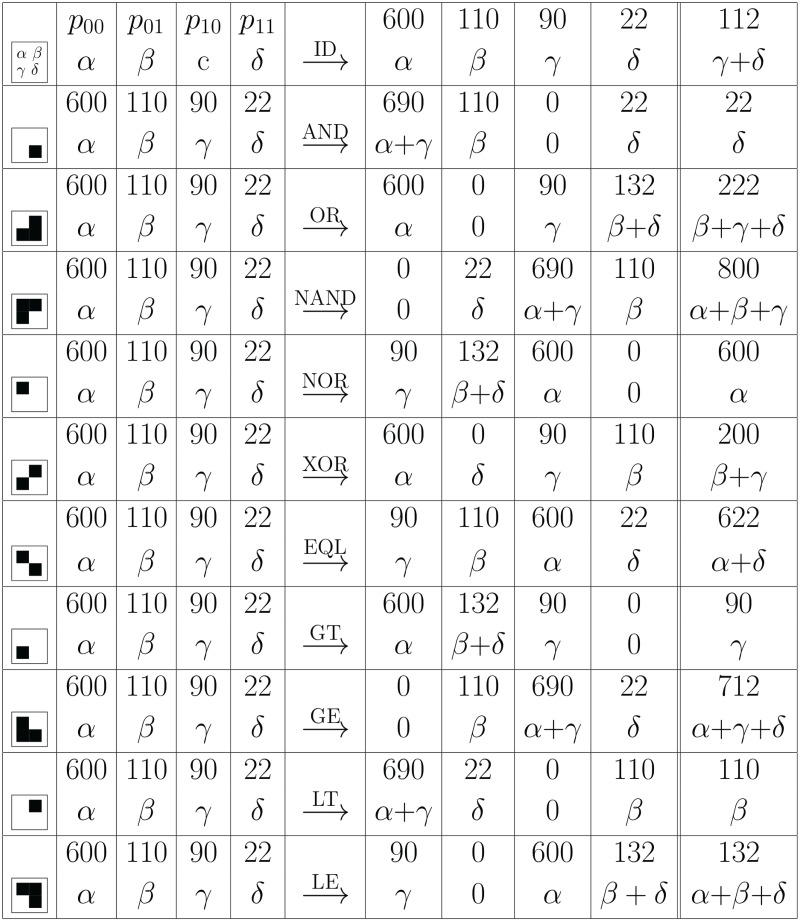
Boolean co-abundance patterns and logical operations. We observe how the abundances of microbe *j*_1_ is shifted by the application of a Boolean operation applied to the abundance pair *p*_00_(*j*_1_, *j*_2_) ≕ *p*_00_ ≕ *α*. Here we use abbreviations *α* ≔ *p*_00_, etc., as shown in the first line. Hence, for each set of samples, the values *α*, *β*, *γ*, *δ* denotes how often each of the four possible configurations occurs. As the result of the Boolean operation replaces *j_i_*, the abundance of *j*_1_ is given by the sum shown in the last column (= sum of previous two columns). For illustration we have included numerical values of co-abundances among 822 samples where *j*_1_ and *j*_2_ are measured 112 and 132 times, resp., and one finds co-occurence of *j*_1_ and *j*_2_ in 22 probes. Here ID denotes the identity operation, AND, OR and XOR (eXclusive-OR) are the common Boolean operations with NAND, NOR and EQL (check if equal) are their logical complements. The operations GT (greater than), GE (greater or equal), LT (less than) and LE (less or equal) are asymmetric comparison operations (see Table Fig 9 for the remaining operations where the output ignores one or two arguments). As one can see, several logical operations lead to visible changes in the *j*_1_ abundance.

Not looking at pairs (i.e. ignoring the second argument) gives us the abundances of *j*_1_ (or *j*_2_) given by *α* + *δ* (or *β* + *δ*), respectively. Now the AND operation shifts the *γ* entries into the *α* field (see Fig 8) which means that the abundance entry for *j*_1_ is shifted to 0 + *δ*. Conversely, the OR operation shifts the *β* entries such they are added to the *δ* entry, such that the abundance entry for *j*_1_ is shifted to *β* + *γ* + *δ*.

Hence the AND operation always leads to an increase of the abundance whereby the OR operation leads to a decrease (or to no change).

**Fig 9:**
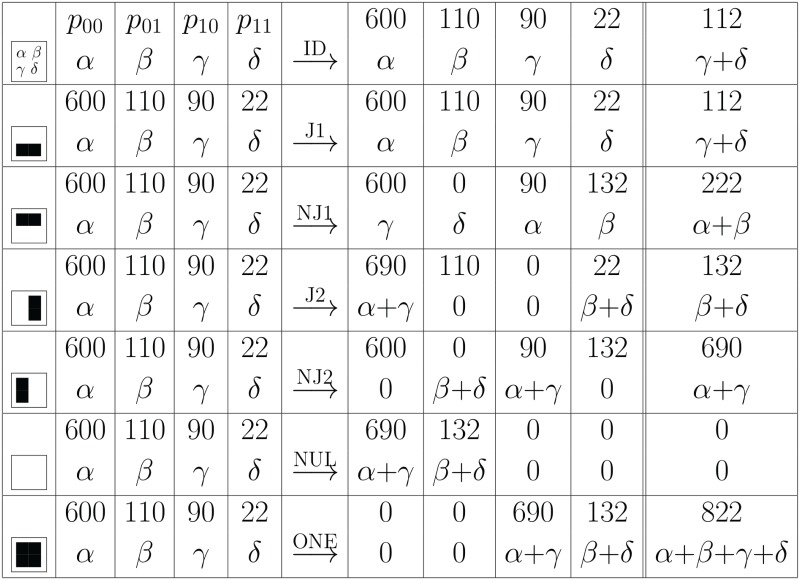
Boolean co-abundance patterns and logical operations. The remaining six possible Boolean operations - here shown for completeness - do not provide any further information. Copying the first entry into the first entry (j1) is the identity operation and leads to no change at all. Copying the j2 entry into j1 leads to a change but only copies existing abundance information. Setting the output bit always to one (last row) conveys zero information such that the output is simply number of samples *α*, *β*, *γ*, *δ*. The other three operations are just logical complements thus likewise convey no additional information.

In summary, the pair abundances before and after the logical shift are given by

$$\begin{array}{ccc} \alpha & ≔ & p_{00}\left(b_{k}^{\left(i\right)}=0,b_{k}^{\left(j\right)}=0\right)\cdot N_{A} \end{array}$$ (4)

$$\begin{array}{ccc} \beta & ≔ & p_{01}\left(b_{k}^{\left(i\right)}=0,b_{k}^{\left(j\right)}=1\right)\cdot N_{A} \end{array}$$ (5)

$$\begin{array}{ccc} \gamma & ≔ & p_{10}\left(b_{k}^{\left(i\right)}=1,b_{k}^{\left(j\right)}=0\right)\cdot N_{A} \end{array}$$ (6)

$$\begin{array}{ccc} \delta & ≔ & p_{11}\left(b_{k}^{\left(i\right)}=1,b_{k}^{\left(j\right)}=1\right)\cdot N_{A} \end{array}$$ (7)

$$\begin{array}{ccc} \tilde{\alpha } & ≔ & p_{00}\left(x_{k}^{\left(i\text{AND}j\right)}=0,b_{k}^{\left(j\right)}=0\right)\cdot N_{A} \end{array}$$ (8)

$$\begin{array}{ccc} \tilde{\beta } & ≔ & p_{10}\left(x_{k}^{\left(i\text{AND}j\right)}=0,b_{k}^{\left(j\right)}=1\right)\cdot N_{A} \end{array}$$ (9)

$$\begin{array}{ccc} \tilde{\gamma } & ≔ & p_{01}\left(x_{k}^{\left(i\text{AND}j\right)}=1,b_{k}^{\left(j\right)}=0\right)\cdot N_{A} \end{array}$$ (10)

$$\begin{array}{ccc} \tilde{\delta } & ≔ & p_{11}\left(x_{k}^{\left(i\text{AND}j\right)}=1,b_{k}^{\left(j\right)}=1\right)\cdot N_{A}, \end{array}$$ (11)

respectively. Here the abundance vector $a_{k}^{\left(i\right)}$ of species *i* is replaced by the shifted abundance vector $x_{k}^{\left(i\right)}$ obtained by entry-wise applying the logical operation AND

$$\begin{array}{c} x_{k}^{\left(i\text{AND}j\right)}≔b_{k}^{\left(i\right)}\text{AND}b_{k}^{\left(j\right)}, \end{array}$$ (12)

similarly for all other possible operations, as shown in Figs 8 and 9 for a concrete example.

These analytical considerations help us better understand the statistical signal extracted from co-abundance data via the ESABO method. As a next step, the entropy shifts will be computed explicitly.

#### Calculating z-scores and entropies from the abundance densities

To compare whether the co-abundances and entropies are significant we calculate their z-scores. For the z-score here we utilize its simple expression from the binomial distribution. For the co-abundance *α*/(*α* + *β* + *γ* + *δ*) we have to rearrange *p*_1_ · *N* = *c* + *d* items within *p*_2_ · *N* = *b* + *d* slots hence its expectation value is *p*_1_ · *p*_2_ · *N* and the variance reads

$$\begin{array}{c} \left(\gamma +\delta \right)\cdot \frac{\left(\beta +\delta \right)}{\alpha +\beta +\gamma +\delta }\cdot \frac{\left(\alpha +\gamma \right)}{\alpha +\beta +\gamma +\delta } \end{array}$$ (13)

For the example shown in Fig 8 the measured co-abundance is 22/822, its expectation value is (90 + 22) · (110 + 22)/822 = 17.99 and the variance reads (90 + 22) · (110 + 22) · (600 + 90)/822^2^ = 15.1 leading to a z-score of (22 − 17.99)/15.1 = 0.26. The z-scores for highest and lowest co-abundances in our dataset are reported in result section *Co-abundance networks: positive and negative interactions*.

As on the relative abundance interval [0, 1] the entropy changes on *o*(1) scale whereas the variance of the binomial distribution narrows down ∼1/*N* with increasing number *N* of samples, the variance of the entropy at density *p*_*i*_ is approximated by $\delta H\left(p_{i}\right)=\delta p_{i}\cdot \frac{\partial H}{\partial p_{i}}$. As a consequence, within low-abundant (resp. high-abundant) species the ranking order of most significants z-scores translates to a respective ranking in the z-scores of entropies.

The entropies are shifted from

$$\begin{array}{ccc} H & = & -\frac{1}{\ln2}\left(-\frac{\gamma +\delta }{\alpha +\beta +\gamma +\delta }\ln\frac{\gamma +\delta }{\alpha +\beta +\gamma +\delta }\right) \end{array}$$ (14)

to

$$\begin{array}{ccc} \tilde{H} & = & -\frac{1}{\ln2}\left(-\frac{\tilde{\delta }}{\alpha +\beta +\gamma +\delta }\ln\frac{\tilde{\delta }}{\alpha +\beta +\gamma +\delta }\right) \end{array}$$ (15)

for the logical AND operation (as the normalization $\alpha +\beta +\gamma +\delta =\tilde{\alpha }+\tilde{\beta }+\tilde{\gamma }+\tilde{\delta }$ is not changed, the denominator remains the same). For the other logical operations, $\tilde{\delta }$ has to be replaced by the respective entries in the last column in Figs 8 or 9. In this way, the entropies can be calculated directly from the co-abundances.

## Supporting information

S1 Code ESABO source code. (TXT)

S1 Text Supporting information. (PDF)
