# Supplementary material for: Boolean analysis reveals systematic interactions among low-abundance species in the human gut microbiome
Source: PLoS Comput Biol. 2017 Jun 22;13(6):e1005361. doi: 10.1371/journal.pcbi.1005361 (PMC5480827; doi:10.1371/journal.pcbi.1005361)
Supplement: S1 Text — (PDF) [file pcbi.1005361.s002.pdf]

Supporting Information

Claussen, Skiecevicene, Wang, Rausch, Karlsen, Lieb, Baines, Franke and Hütt
PLoS Comp Biol 2017 doi: insertDOIhere

Detailed numerical results of the ESABO analysis for human gut microbiome compositions

Investigation of the influence of the binarization threshold on presence/absence patterns of microbial species

Here we re-computed the interaction networks also for a threshold of 2 (i.e., abundance values of 0 and 1 are set to 0; all others are set to 1). This yields a systematically lower number of links, as can be seen in Figure A, which shows the number of links as a function of the z-score threshold. Hence the binarization threshold of 1 (i.e., any non-zero abundance is set to 1) is an appropriate choice.

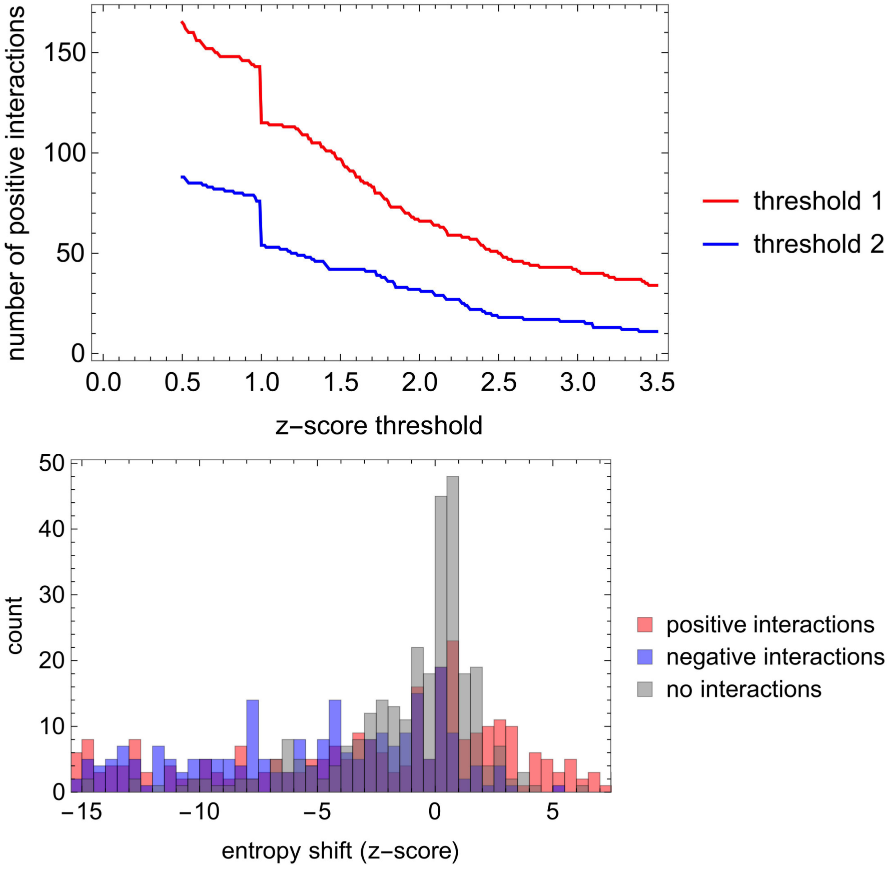

Figure A: Number of predicted positive interactions (upper part) and predicted negative interactions (lower part) as a function of the threshold in the entropy shift z-score. Red curves: binarization threshold 1, blue curves: binarization threshold 2.

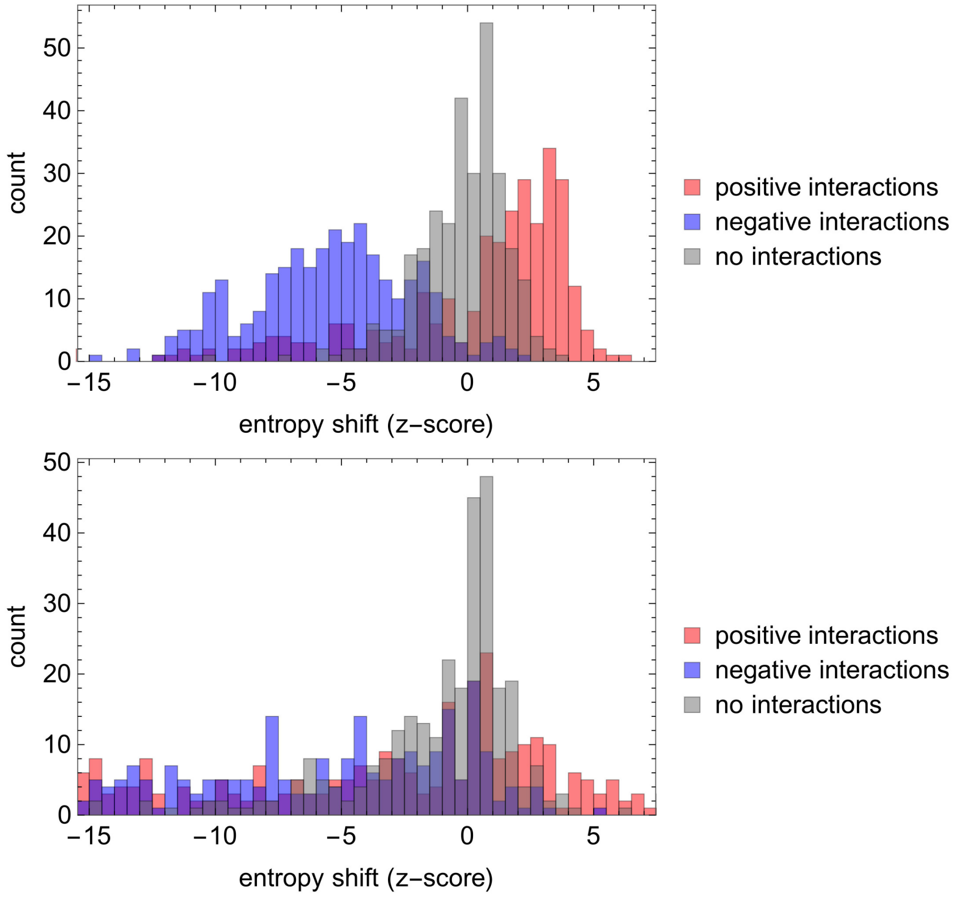

Figure B: Histograms of z-scores of entropy shifts obtained with the ESABO method (Boolean AND) applied to simulated binary abundance patterns from 20 species interaction networks consisting of 15 nodes and 15 positive and negative interactions, respectively. Blue: negative interactions, red: positive interactions, gray: random sample of absent links. Upper histogram: all attractors are sampled uniformly; lower histogram: attractors are sampled according to their basin size. The upper histogram is similar to the one shown in Figure 2 (Note that 'mixed colors' appear, when histograms overlap.)

Histogram of z-scores for 15 positive and 15 negative interactions

Figure B shows that sampling attractors according to their basin size reduces the prediction quality of the links.

Figure C displays the percentage of fixed point attractors as a function of the number of links.

One of the limitations of the Boolean model used to simulate (binary) abundance patterns from species interaction networks is that we are limited in the range of connectivities producing a substantially rich set of abundance patterns (i.e. attractors). Figure D shows the number of attractors as a function of connectivity for species interaction networks with 15 nodes for three different schemes of changing connectivity. Due to the rapid decrease of the number of attractors with connectivity in all three schemes, we restrict to the choice $M_{-} = const = 15$ and $M_{+} = const = 15$.

Regarding our choice of parameters, two points should be noted: (1) There is no reliable *a priori* information about suitable ranges of connectivity. In particular, one of the main findings of the present investigation is that the dominant (and well known) interactions among high-abundance species are embedded in a large network of (mostly positive) interactions of

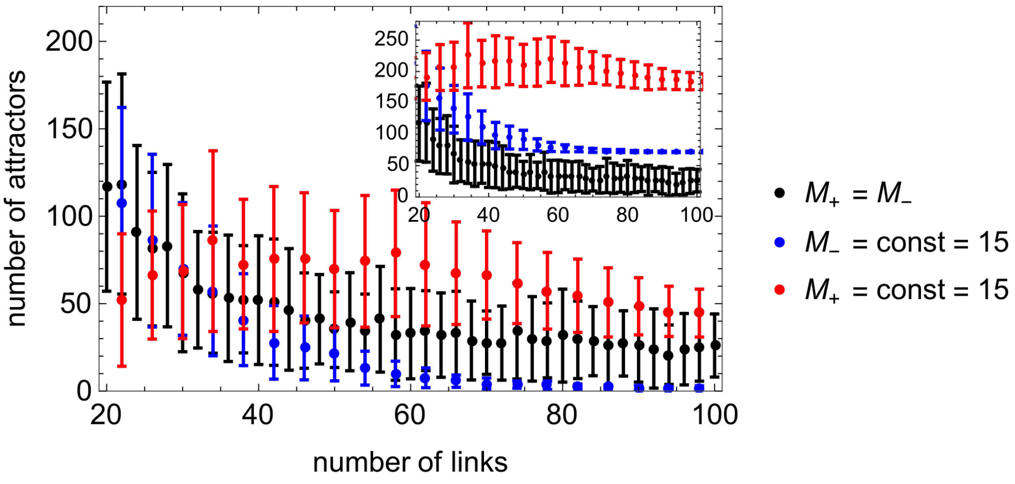

Figure C: Percentage of fixed point attractors as a function of the number of links for simulated species interaction networks ($N = 15$) for different connectivity schemes: $M_+ = M_I$ (black), $M_- = const = 15$ (blue) and $M+ = const = 15$. Averages and standard deviations (error bars) are obtained from 40 runs for each connectivity. For a better view of the individual curves, the inset shows the same curves with the blue and the red curves shifted downwards by 0.3 and 0.6, respectively.

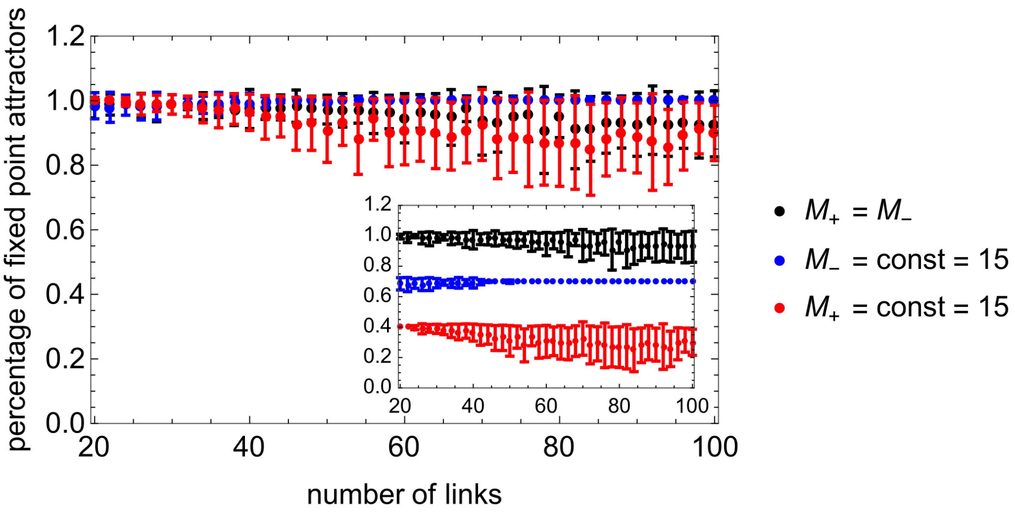

Figure D: Number of attractors as a function of the number of links for simulated species interaction networks ($N = 15$) for different connectivity schemes: $M_+ = M_I$ (black), $M_- = const = 15$ (blue) and $M+ = const = 15$. Averages and standard deviations (error bars) are obtained from 40 runs for each connectivity. For a better view of the individual curves, the inset shows the same curves with the blue and the red curves shifted upwards by 70 and 140, respectively.

low-abundance species. (2) Our choice of requiring more than 100 distinct attractors is purely heuristic. It is intuitively clear that the quality should decrease with decreasing numbers of attractors. However, we have not studied this decrease in detail.

The prediction quality is, to a certain extent, arbitrary, as it is based on specific thresholds: We count as correctly classified the cases, where the Jaccard index was larger than 0.6 and as incorrectly classified (thus counting negatively) those cases, where the Jaccard index was smaller than 0.4. Figure E shows a histogram of prediction qualities for the Boolean AND and for the Jaccard index for 80 simulated species interaction networks ($N = 15$, $M_+ = M_- = 15$) at a binary noise level of $p = 0.2$ and a threshold for the Jaccard index of 0.8. It is quite clear that for this choice of parameters the Boolean AND performs substantially better in recovering positive interactions. Based on a wide range of such simulations, we are convinced that the Boolean analysis is the better choice, if connectivity and noise levels are not *a priori* known. We also intend to extend the ESABO analysis further by allowing the choice, whether binarized abundance vectors are evaluated via the entropy shifts based on (several) Boolean operations or via the Jaccard index.

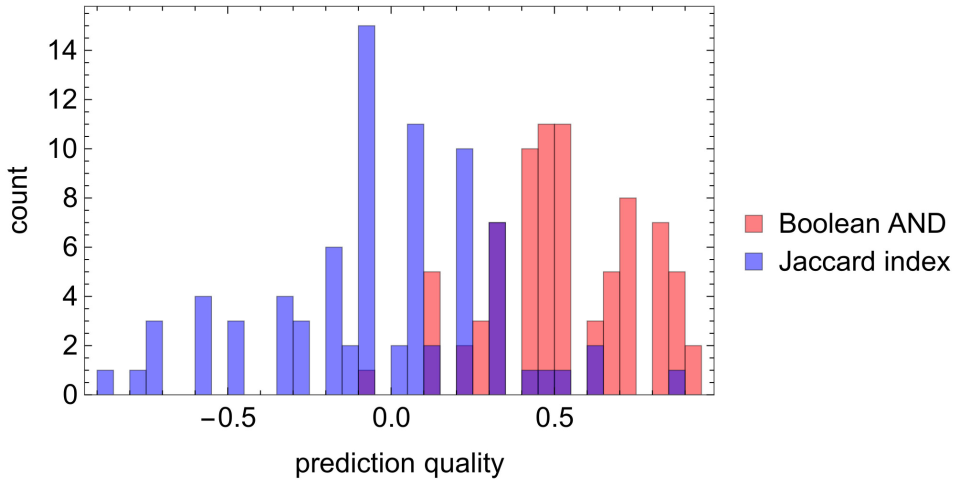

Figure E: Histogram of prediction qualities for positive interactions based on 80 simulated species interaction networks ($N = 15$, $M_+ = M_- = 15$) at a binary noise level of $p = 0.2$. Blue: prediction quality obtained from the Jaccard index (detection threshold 0.8: If the Jaccard index for a positive interaction is 0.8 or higher, the link is counted as 'correctly predicted'; if it is below 0.8 it is counted as 'incorrectly predicted'). Red: prediction quality obtained from ESABO (Boolean AND). (Note that 'mixed colors' appear, when histograms overlap.)

Given the binarized abundance data, there are two additional filtering steps, which may be applied: (1) Eliminating dublicates from the abundance vectors (filter $\mathcal{D}$). In the analysis of the simulated abundance data, this step has significantly improved the detection quality (cf. Figure S2). (2) Discarding taxa with near-constant abundance vectors (i.e. taxa that are either almost always present or almost always absent; filter $\mathcal{C}$). Table A shows how many steady state abundance vectors and how many taxa remain after each of these steps and how many positive and negative links the ESABO analysis yields, when applied with and without these filtering steps. The overall picture emerging from applying the ESABO analysis to different taxonomic levels is that there is a substantial number of significant interactions and that the positive interactions tend to be much more frequent than the negative interactions.
As pointed out above, the due to the binarization, the ESABO analysis rather focuses on the low-abundance species. The multi-level analysis summarized in Table A thus supports our key result presented in section *Analysis of the human gut microbiome compositions*, namely

Table A: Summary of ESABO results on different taxonomic levels. In all cases, a Boolean AND and z-score thresholds of ±1 have been used. Furthermore, the filtering of near-constant vectors (filter $\mathcal{C}$) required vectors to have at least three zeros and three ones, in oder to enter the ESABO analysis.

level	filter	steady states	taxa	M_+	M_-
**phyla**	none	822	26	54	4
	$\mathcal{D}$	78	26	26	19
	$\mathcal{D}$, $\mathcal{C}$	78	13	15	17
**class**	none	822	42	179	22
	$\mathcal{D}$	393	42	146	35
	$\mathcal{D}$, $\mathcal{C}$	393	24	112	29
**order**	none	822	77	518	52
	$\mathcal{D}$	527	77	443	74
	$\mathcal{D}$, $\mathcal{C}$	527	40	293	59

that the well-known, strong (mostly inhibitory) links in microbial interaction networks are embedded in a dense systematic network of (mostly positive) interactions among low-abundance species.
For the detailed phyla-level analysis presented in section *Analysis of the human gut microbiome compositions*, we opted for the unfiltered version, as the data matrix becomes indeed very small under these filtering steps.

Definition of prediction quality

The prediction quality for the ESABO score shown in Figure 4 is computed using the following 'template': (number of successful prediction − number of unsuccessful predictions)/(total number of cases in this category). For positive interactions:

$$Q_+^{(z)} = (|z > 1| - |z < -1|)/M_+,$$

where, e.g., $|z > 1|$ denotes the number of times (out of the M_+ positive interactions) a z-score larger than 1 has been found. For negative interactions:

$$Q_-^{(z)} = (|z < -1| - |z > 1|)/M_-.$$

The Jaccard index is defined on the level of pairs of binary values in two binary vectors a and b. For positive interactions:

$$J_1(a, b) = \#(1, 1)/\min[\#1(a), \#1(b)],$$

where $\#(1, 1)$ denotes the number of components in a and b where a 1 in a and a 1 in b occur simultaneously. Correspondingly, $\#1(a)$ denotes the total number of 1s in vector a. For negative interactions the Jaccard index is evaluated on the level of pairs of zeros:

$$J_0(a, b) = \#(0, 0)/\min[\#0(a), \#0(b)].$$

The prediction quality for the Jaccard indices in the case of positive interactions is:

$$Q_+^{(J)} = (|J_1 > 0.6| - |J_1 < 0.4|)/M_+$$

and the corresponding equation for negative interactions.

Abundance and co-abundance tables

Table B: The 25 classified phyla in our dataset labeled 1 ... 25. Unclassified Bacteria are labeled with 26. The last column displays in how many of the 822 samples a bacterium from the phyla is detected.

Label	Phylum	Abundance
1	Acidobacteria	57
2	Actinobacteria	585
3	Aquificae	65
4	Bacteriodetes	822
5	Chlorobi	1
6	Chloroflexi	1
7	Chrysiogenetes	51
8	Cyanobacteria	102
9	Deferribacteres	2
10	Deinococcus-Thermus	6
11	Firmicutes	822
12	Fusobacteria	36
13	Gemmatimonadetes	6
14	Lentisphaerae	16
15	Nitrospira	17
16	OP10	1
17	Planctomycetes	4
18	Proteobacteria	779
19	Spirochaetes	10
20	Synergistetes	37
21	TM7	27
22	Tenericutes	306
23	Thermodesulfobacteria	4
24	Verrucomicrobia	136
25	WS3	1
26	unclassified-Bacteria	137

Table C: Table of lowly co-abundant phyla (z-score ≤ -1.0).

j_1	j_2	$a(0,0)$	$a(0,1)$	$a(1,0)$	$a(1,1)$	$pred[a(1,1)]$	$vari(1,1)$	$zscore(1,1)$
2	6	236	1	585	0	0.71	0.71	-1.00
26	17	681	4	137	0	0.67	0.66	-1.00
24	10	680	6	136	0	0.99	0.99	-1.01
7	14	755	16	51	0	0.99	0.97	-1.02
12	14	770	16	36	0	0.70	0.69	-1.02
21	14	779	16	27	0	0.53	0.52	-1.02
21	15	778	17	27	0	0.56	0.55	-1.02
14	21	779	27	16	0	0.53	0.51	-1.03
15	21	778	27	17	0	0.56	0.54	-1.03
14	12	770	36	16	0	0.70	0.67	-1.05
14	7	755	51	16	0	0.99	0.93	-1.07
10	24	680	136	6	0	0.99	0.83	-1.20
17	26	681	137	4	0	0.67	0.56	-1.20
6	2	236	585	1	0	0.71	0.21	-3.47

Table D: Table of highly co-abundant phyla (zscore ≥ 1.5).

j_1	j_2	$a(0,0)$	$a(0,1)$	$a(1,0)$	$a(1,1)$	$pred[a(1,1)]$	$vari(1,1)$	$zscore(1,1)$
14	20	774	32	11	5	0.72	0.69	6.22
20	14	774	11	32	5	0.72	0.71	6.06
10	8	718	98	2	4	0.74	0.65	4.99
15	20	772	33	13	4	0.77	0.73	4.43
8	10	718	2	98	4	0.74	0.74	4.40
20	15	772	13	33	4	0.77	0.75	4.32
19	1	758	54	7	3	0.69	0.65	3.57
1	19	758	7	54	3	0.69	0.68	3.37
21	1	745	50	20	7	1.87	1.74	2.94
1	21	745	20	50	7	1.87	1.81	2.83
19	22	516	296	0	10	3.72	2.34	2.69
15	26	677	128	8	9	2.83	2.36	2.61
19	8	714	98	6	4	1.24	1.09	2.54
13	24	683	133	3	3	0.99	0.83	2.42
19	24	681	131	5	5	1.65	1.38	2.42
19	7	763	49	8	2	0.62	0.58	2.37
7	19	763	8	49	2	0.62	0.61	2.25
8	19	714	6	98	4	1.24	1.23	2.25
26	15	677	8	128	9	2.83	2.77	2.22
15	3	744	61	13	4	1.34	1.24	2.15
24	19	681	5	131	5	1.65	1.63	2.05
24	13	683	3	133	3	0.99	0.99	2.04
3	15	744	13	61	4	1.34	1.32	2.02
12	1	736	50	29	7	2.50	2.32	1.94
13	8	716	100	4	2	0.74	0.65	1.93
15	22	513	292	3	14	6.33	3.97	1.93
20	24	665	120	21	16	6.12	5.11	1.93
20	26	664	121	21	16	6.17	5.14	1.91
1	12	736	29	50	7	2.50	2.39	1.89
20	22	509	276	7	30	13.77	8.65	1.88
22	19	516	0	296	10	3.72	3.68	1.71
8	13	716	4	100	2	0.74	0.74	1.70
24	20	665	21	120	16	6.12	5.85	1.69
3	26	646	111	39	26	10.83	9.03	1.68
19	26	679	133	6	4	1.67	1.39	1.68
26	20	664	21	121	16	6.17	5.89	1.67
15	1	751	54	14	3	1.18	1.10	1.66
19	3	749	63	8	2	0.79	0.73	1.66
17	22	515	303	1	3	1.49	0.93	1.62
23	22	515	303	1	3	1.49	0.93	1.62
21	12	762	33	24	3	1.18	1.13	1.61
12	21	762	24	33	3	1.18	1.14	1.59
1	15	751	14	54	3	1.18	1.15	1.58
3	22	499	258	17	48	24.20	15.19	1.57
3	19	749	8	63	2	0.79	0.78	1.55
26	3	646	39	111	26	10.83	9.98	1.52
20	3	727	58	30	7	2.93	2.69	1.51

Table E: (Continued.) Table of highly co-abundant phyla (1.5 ≥ zscore ≥ 1.0).

j_1	j_2	$a(0,0)$	$a(0,1)$	$a(1,0)$	$a(1,1)$	$pred[a(1,1)]$	$vari(1,1)$	$zscore(1,1)$
3	20	727	30	58	7	2.93	2.79	1.46
26	19	679	6	133	4	1.67	1.65	1.42
5	2	237	584	0	1	0.71	0.21	1.41
9	2	237	583	0	2	1.42	0.41	1.41
10	2	237	579	0	6	4.27	1.23	1.41
13	2	237	579	0	6	4.27	1.23	1.41
16	2	237	584	0	1	0.71	0.21	1.41
17	2	237	581	0	4	2.85	0.82	1.41
19	2	237	575	0	10	7.12	2.05	1.41
23	2	237	581	0	4	2.85	0.82	1.41
25	2	237	584	0	1	0.71	0.21	1.41
14	22	511	295	5	11	5.96	3.74	1.35
10	22	514	302	2	4	2.23	1.40	1.26
20	7	739	46	32	5	2.30	2.15	1.26
26	22	470	215	46	91	51.00	32.01	1.25
21	8	700	95	20	7	3.35	2.93	1.24
22	15	513	3	292	14	6.33	6.20	1.24
7	20	739	32	46	5	2.30	2.19	1.23
22	20	509	7	276	30	13.77	13.15	1.23
12	3	727	59	30	6	2.85	2.62	1.20
3	12	727	30	59	6	2.85	2.72	1.16
8	21	700	20	95	7	3.35	3.24	1.13
22	3	499	17	258	48	24.20	22.28	1.07
3	18	43	714	0	65	61.60	3.22	1.06
5	18	43	778	0	1	0.95	0.05	1.06
6	18	43	778	0	1	0.95	0.05	1.06
7	18	43	728	0	51	48.33	2.53	1.06
7	26	650	121	35	16	8.50	7.08	1.06
9	18	43	777	0	2	1.90	0.10	1.06
10	18	43	773	0	6	5.69	0.30	1.06
13	18	43	773	0	6	5.69	0.30	1.06
14	18	43	763	0	16	15.16	0.79	1.06
14	24	675	131	11	5	2.65	2.21	1.06
16	18	43	778	0	1	0.95	0.05	1.06
17	18	43	775	0	4	3.79	0.20	1.06
19	18	43	769	0	10	9.48	0.50	1.06
23	18	43	775	0	4	3.79	0.20	1.06
25	18	43	778	0	1	0.95	0.05	1.06
14	26	674	132	11	5	2.67	2.22	1.05
20	1	733	52	32	5	2.57	2.39	1.02
22	17	515	1	303	3	1.49	1.48	1.02
22	23	515	1	303	3	1.49	1.48	1.02

Table F: Table of phyla where an entropy shift (ESABO score) detects (with $|\mathrm{z-score}| \geq 1$) an inhibitory or activating co-abundance.

```
inhibitory:
2 6 -1.49466
2 18 -1.27253
7 14 -1.12893
10 24 -1.21224

activating:
1 2 2.15874
1 3 1.62298
1 8 1.96282
1 10 6.18977
1 12 2.69013
1 13 6.34225
1 15 1.79631
1 16 3.56173
1 17 6.19778
1 18 1.21151
1 19 2.7535
1 20 1.59899
1 21 3.40927
1 22 1.37597
1 23 3.16231
1 24 1.67712
2 3 1.59495
2 8 1.24849
2 10 1.46656
2 13 1.51621
2 17 1.2567
2 19 1.94983
2 20 2.41183
2 21 2.07411
2 22 1.31233
2 23 1.23677
2 24 2.26892
2 26 2.49806
3 7 1.54251
3 9 2.16431
3 12 1.77778
3 15 2.17819
3 17 1.39182
3 18 1.81795
3 19 1.40844
3 20 2.37135
3 21 1.37929
3 22 5.6536
3 24 1.71019
3 26 4.43228
6 22 1.29035
6 24 2.40768
7 8 1.13819
7 15 1.04411
7 18 1.71236
7 19 1.8868
7 20 1.75151
7 22 3.19134
7 24 1.71405
7 26 2.52974
8 9 1.69379
8 10 3.53032
8 12 1.76503
8 13 1.6435
8 16 2.65659
8 17 2.12716
8 18 1.55202
8 19 2.44218
8 21 1.99586
8 22 1.29606
8 23 2.31277
9 12 2.96065
9 21 3.44559
10 12 3.56834
10 13 4.65892
10 16 12.8647
10 17 6.00008
10 21 5.56994
10 22 1.44616
10 23 11.9822
12 21 1.62652
12 23 1.90055
13 16 11.13
13 17 10.2587
13 19 3.62602
13 20 1.58444
13 21 5.63558
13 23 5.55944
13 24 2.08574
14 15 4.27423
14 19 1.93504
14 20 4.48393
14 22 2.58167
14 24 1.50654
14 26 1.51676
15 19 1.80898
15 20 3.23734
15 22 3.52253
15 24 1.47717
15 26 3.42929
16 17 15.7718
16 21 5.3276
16 23 18.2209
17 19 3.88056
17 20 1.89004
17 21 4.61476
17 22 1.46339
17 23 7.18192
19 20 2.36041
19 22 3.55012
19 24 2.55917
19 25 9.9449
19 26 1.80349
20 22 5.09416
20 24 3.81434
20 25 4.89653
20 26 3.82695
21 23 2.5139
22 23 1.52192
22 24 2.99716
22 25 1.31833
22 26 6.66629
24 25 2.17015
24 26 3.01985
25 26 2.38866
```

Table G: Co-abundances of phyla: Table of correlation coefficient for pairs (larger than 0.4 or smaller than -0.10) of phyla. The third column lists the Pearson correlation coefficient c_{ij} for the pairs of phyla with the numbers i and j displayed in columns 1 and 2. As expected, phyla 4 and 11 display a strong anticorrelation of -0.892683 and the two pairs 2 - 4 and 11 - 18 show a small anticorrelation. On the side of positive correlations there are nine stronger co-abundances whereof three (1-16, 1-17 and 1-13) have a correlation coefficient ≥ 0.5.

1	16	0.683096
1	17	0.655906
1	13	0.561508
16	17	0.499086
16	23	0.499086
9	21	0.479777
10	16	0.473349
13	16	0.407003
13	17	0.404751
...		
11	12	-0.102470
1	4	-0.106288
4	18	-0.121335
4	22	-0.148210
11	18	-0.182161
2	4	-0.215102
4	11	-0.892683

Re-analysis of the American Gut dataset

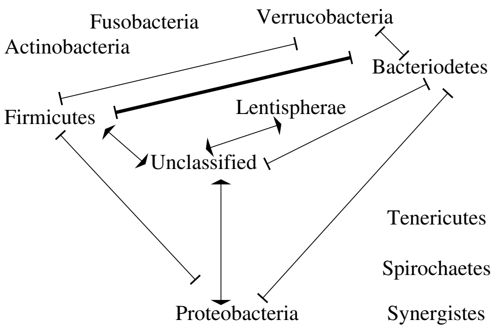

Figure F: Links of the phyla interaction network, as compiled for the within-gut interactions from the data from Faust et al. [12]. The network is comprised by three positive weak interactions, one strong inhibiting link (between Bacteriodetes and Firmicutes) and five weak negative interactions.

Table H: Cumulated link strengths for the network shown in Fig. F

Bacteriodetes	Firmicutes	−116
Verrucomicrobia	Bacteriodetes	−8
Proteobacteria	Bacteriodetes	−4
Proteobacteria	Firmicutes	−4
Verrucomicrobia	Firmicutes	−4
Bacteriodetes	Unclassified	−9
Firmicutes	Unclassified	+4
Lentispherae	Unclassified	+2
Proteobacteria	Unclassified	+2

ESABO source code

The following Mathematica code is the core of the code used for the ESABO calculation. It can be easily adapted to other programming languages.

```
simpleLog[x_] := If[x == 0, 10^-5, Log[x]]
entropyTemp[vec_] := Module[{t1, t2, t3},
  t1 = Length[vec];
  t2 = N[Count[vec, 0]/t1];
  t3 = N[Count[vec, 1]/t1];
  - (t2 * simpleLog[t2] + t3* simpleLog[t3])]
shiftScore[twovec_, op_, rep_] := Module[{x1, x2, x3, x4, x5},
  x1 = Map[op[Sequence @@ #] &, twovec];
  x2 = entropyTemp[x1];
  x3 = Table[
    entropyTemp[
     Map[op[Sequence @@ #] &,
      Transpose[Map[RandomSample, Transpose[twovec]]]]], {rep}];
  x4 = N[Mean[x3]];
  x5 = N[StandardDeviation[x3]];
  If[x5 == 0, 0, (x2 - x4)/x5]]
evaluateESABO[filename_, random_, booleanOp_, threshold_] :=
 Module[{y1, y2, y3, y4, y5, y6, y7},
  y1 = Import[filename, "Data"];
  y2 = Map[StringSplit[#, " "] &, y1];
  y3 = Drop[y2, 1][[All, 2 ;; Length[y2[[2]]]]];
  y4 = Map[ToExpression, y3];
  y5 = y4 /. ( n1_Integer ) :> If[n1 > (threshold - 1), 1, 0];
  y6 = Table[{y2[[1]][[i]], y2[[1]][[j]],
     shiftScore[Thread[{y5[[All, i]], y5[[All, j]]}], booleanOp,
      random]}, {i, 1, Length[y2[[1]]]}, {j, i + 1, Length[y2[[1]]]}]]
```

```
Herein the arguments of ESABO are

filename:  defines the filename including the path
random:  defines the number of randomized vectors for calculation of the z-scores
booleanOp:  specifies the boolean operation (hier: AND)
threshold:  sets the binarization threshold of the abundance data

The main results (pairs of species names, entropy shift z-score) then were obtained by

evaluateESABO["phyla_1000.otu_matrix_row", 1000, BitAnd, 1]
```
